# First Evidence That Nematode Communities in Deadwood Are Related to Tree Species Identity and to Co-Occurring Fungi and Prokaryotes

**DOI:** 10.3390/microorganisms9071454

**Published:** 2021-07-06

**Authors:** Julia Moll, Friederike Roy, Claus Bässler, Jacob Heilmann-Clausen, Martin Hofrichter, Harald Kellner, Doris Krabel, Jan Henrik Schmidt, François Buscot, Björn Hoppe

**Affiliations:** 1 Helmholtz Centre for Environmental Research—UFZ, Department of Soil Ecology, 06120 Halle (Saale), Germany; friederike.roy@gmx.de (F.R.); francois.buscot@ufz.de (F.B.); 2 Institute of Forest Botany, Technische Universität Dresden, 01737 Tharandt, Germany; doris.krabel@tu-dresden.de; 3 Department of Conservation Biology, Institute of Ecology, Evolution and Diversity, Goethe University Frankfurt, 60438 Frankfurt am Main, Germany; baessler@bio.uni-frankfurt.de; 4 Department of Research, National Park Bavarian Forest, 94481 Grafenau, Germany; 5 Center for Macroecology, Evolution and Climate, GLOBE Institute, University of Copenhagen, 2100 Copenhagen, Denmark; jheilmann-clausen@sund.ku.dk; 6 Institute of Environmental Biotechnology, Technische Universität Dresden, IHI Zittau, 02763 Zittau, Germany; martin.hofrichter@tu-dresden.de (M.H.); harald.kellner@tu-dresden.de (H.K.); 7 Institute for Epidemiology and Pathogen Diagnostics, Julius Kühn Institute (JKI)—Federal Research Centre for Cultivated Plants, 38104 Braunschweig, Germany; jan-henrik.schmidt@julius-kuehn.de; 8 German Centre for Integrative Biodiversity Research (iDiv) Halle—Jena—Leipzig, 04103 Leipzig, Germany; 9 Institute for National and International Plant Health, Julius Kühn Institute (JKI)—Federal Research Centre for Cultivated Plants, 38104 Braunschweig, Germany

**Keywords:** amplicon sequencing, bacteria, decomposition, metabarcoding, nematode diversity, temperate forest, trophic interactions

## Abstract

Nematodes represent a diverse and ubiquitous group of metazoans in terrestrial environments. They feed on bacteria, fungi, plants, other nematodes or parasitize a variety of animals and hence may be considered as active members of many food webs. Deadwood is a structural component of forest ecosystems which harbors many niches for diverse biota. As fungi and bacteria are among the most prominent decomposing colonizers of deadwood, we anticipated frequent and diverse nematode populations to co-occur in such ecosystems. However, knowledge about their ability to colonize this habitat is still limited. We applied DNA-based amplicon sequencing (metabarcoding) of the 18S rRNA gene to analyze nematode communities in sapwood and heartwood of decaying logs from 13 different tree species. We identified 247 nematode ASVs (amplicon sequence variants) from 27 families. Most of these identified families represent bacterial and fungal feeders. Their composition strongly depended on tree species identity in both wood compartments. While pH and water content were the only wood properties that contributed to nematodes’ distribution, co-occurring fungal and prokaryotic (bacteria and archaea) α- and β-diversities were significantly related to nematode communities. By exploring thirteen different tree species, which exhibit a broad range of wood characteristics, this study provides first and comprehensive insights into nematode diversity in deadwood of temperate forests and indicates connectivity to other wood-inhabiting organisms.

## 1. Introduction

The decomposition of deadwood is driven by a broad range of wood-inhabiting biota under the influence of various factors. Besides environmental conditions such as precipitation and temperature, physico-chemical characteristics of the wood itself influence the decomposition process [1,2]. These intrinsic properties such as the content of cellulose, hemicelluloses (xylan, glucomannan) and recalcitrant lignin greatly differ between coniferous and deciduous trees [3]. There are also strong differences between the compartments of a tree (e.g., roots, trunk, branches or leaves) or even within a single trunk—where the inner part of a living tree, so-called heartwood, is functionally dead and mainly acts as a stabilizing element. It is therefore much drier and richer in extractives than the surrounding sapwood that carries water vertically from roots to leaves, and nutrients and other organic molecules horizontally [4]. This causes spatial heterogeneity in decaying logs forming different microhabitats that are occupied by high numbers of arthropods, fungi, bacteria and archaea [5,6,7]. 

Due to their ability to produce a variety of extracellular enzymes which enable the breakdown of complex plant components, fungi and saproxylic arthropods, along with their symbiotic microbes, are main actors of wood decomposition [8,9]. During the last several decades, the contribution and distribution of micro- and macro-fungi during wood decomposition has been intensively investigated, showing that community composition and diversity patterns were linked to land use intensity, host tree identity and corresponding wood physico-chemical properties (e.g., [7,10,11,12]). Besides investigations focusing on fungi and arthropods, recent research efforts have investigated prokaryotic activities in deadwood [13,14,15,16,17,18], concluding that bacteria and also archaea act as protagonists in this complex and diverse ecosystem. Besides these mentioned organisms, which contribute directly to the decomposition process, deadwood also hosts organisms which are not directly capable of utilizing wood as a resource but are anticipated to be members of the overall food web. 

Nematodes represent a diverse group of metazoans that inhabit almost every habitat on Earth. They are prominent soil-inhabitants and appreciated indicators for soil quality and functioning [19]. Their distribution is influenced by abiotic factors such as moisture, pH, temperature and soil characteristics as well as the availability of feeding resources [20,21,22]. Based on their feeding behaviours, nematodes can be basically divided into the following functional groups: fungivores, bacterivores, herbivores, omnivores, predators, and parasites [23]. Hence, they are important actors in aquatic and terrestrial food webs and channel resources to higher trophic levels [24]. In deadwood, fungi and bacteria in particular, but also plant cells derived residual sugars or oligosaccharides, may provide food resources for nematodes. On the other hand, various nematophagous fungi [25], such as wood rot fungi of the genus *Pleurotus*, trap nematodes (e.g., *Poikilolaimus oxycercus*, Rhabditidae) and, in turn, can serve as food for fungivorous nematodes [26,27].

The isolation of living nematodes from wood is widely applied in the field of plant health to monitor the global spread of the plant-parasitic pinewood nematode *Bursaphelenchus xylophilus* [28]. Although this ecologically and economically relevant species and also other members of the genus have been studied comprehensively, we could not identify a single investigation with clear emphasis to overall nematode diversity in (dead) wood. It appears rather unreasonable that wood-inhabiting nematodes, other than plant-parasitic specimen, are largely unexplored, especially as deadwood is an appreciated biodiversity hotspot in forest ecosystems [29,30]. 

While several factors may have contributed to this lacking knowledge, we identified two which are of particular relevance: (i) Importance of deadwood and its significance for biodiversity in forest ecosystems have been largely neglected compared to forest soils; (ii) Due to the microscopic size of nematodes and inconsistent taxon-characteristic features, identification by classic non-molecular methods is rather difficult and requires expert knowledge [31,32]. In addition, research in phytonematology has an emphasis on plant parasitic nematodes over free-living nematodes. Therefore, the introduction of amplicon sequencing to also survey nematodes should provide new opportunities to analyze and compare community structures and spatial distributions in deadwood [33,34,35]. 

The present study reports findings from the BELongDead (Biodiversity Exploratories Long-term Deadwood) experiment that elucidates the decomposition of deadwood logs of thirteen coniferous and deciduous temperate tree species, standardized by the same starting time point of decomposition [36]. During the project, diverse fungal and prokaryotic community structures were observed between tree species and between heartwood and sapwood compartments, which confirmed a high degree of host tree specialization and spatial heterogeneity [37,38,39]. By amplicon-sequencing of the 18S rRNA gene, we here aimed to provide first comprehensive insights into nematode diversity and community structure within this standardized and well-characterized set-up of deadwood logs. Hence, this investigation not only includes extensive data on wood physico-chemical properties, but also on prokaryotes and fungi captured on exactly the same wood samples. We addressed the following research questions: (i) Does nematode community structure differ between host trees and in relation to the respective wood-characteristics, as these are apparently highly variable? (ii) Can variability of nematode community structure be explained by corresponding and connected fungal and prokaryotic community structures and diversity patterns? (iii) Is amplicon sequencing a useful tool to explore nematode diversity in deadwood? 

## 2. Materials and Methods

### 2.1. Study Area and Sampling

The study was performed within the German Biodiversity Exploratories [40]. In late 2008, an experimental platform for exploring the diversity of deadwood-inhabiting biota and corresponding decomposition processes was established, the so-called BELongDead (Biodiversity Exploratories Longterm Deadwood) experiment [36]. Freshly cut logs of 13 temperate tree species were placed in threefold replication on representative research plots under different forest management intensities. The design comprises nine decidious species: maple (*Acer* spp.), birch (*Betula pendula* Roth), hornbeam (*Carpinus betulus* L.), European beech (*Fagus sylvatica* L.), European ash (*Fraxinus excelsior* L.), aspen (*Populus* spp.), wild cherry (*Prunus avium* L.), oak (*Quercus* spp.), and lime tree (*Tilia* spp.), and four coniferous species: European larch (*Larix decidua* Mill.), Norway spruce (*Picea abies* L., H. Karst.), Scots pine (*Pinus sylvestris* L.), and Douglas fir (*Pseudotsuga menziesii* (Mirb.), Franco). Samples were collected from three experimental plots dominated by *Fagus sylvatica* and a standardized forest management practice (selection cutting) at the Nationalpark Hainich in Central Germany (N 51.08, E 10.43). In June 2014, after more than 5 years of exposition, the majority of logs reached the transition from the early to middle stage of decomposition. Bark was partly absent, but the wood largely maintained its color and structure. Distinct sampling of wood in heartwood and sapwood followed a procedure previously described [38,39]. In total, 82 samples (13 tree species × 3 experimental plots × 2 sampling depths + sapwood and heartwood from 2 additional logs) were collected. Prior to further analyses, each wood sample was homogenized into a fine powder under liquid nitrogen using a swing mill (Retsch, Haan, Germany).

### 2.2. DNA Extraction, Nematode PCR and Sequencing

The same genomic DNA already applied for prokaryotic and fungal PCR-based community analyses [38,39] was used for nematode PCRs. These DNA extracts were isolated from 250 mg homogenized wood sample using a ZR Soil Microbe DNA MiniPrep kit (Zymo Research, Irvine, CA, USA) according to the manufacturer’s protocol. The nematode-specific small subunit sequence (SSU) of the ribosomal RNA gene was amplified using a semi-nested PCR procedure of Sapkota and Nicolaisen [34]. First, a fragment of ~520 bp was generated using the primer pair (1) NemF (5′-GGGGAAGTATGGTTGCAAA-3′) and 18Sr2b (5′-TACAAAGGGCAGGGACGTAAT-3′). PCR products were 1/10 diluted and used as template for the second amplification using the primer pair (2) N1F (5′-GGTGGTGCATGGCCGTTCTTAGTT-3′) and 18Sr2b containing Illumina Nextera xt adapter sequences for compatibility with Illumina index adapters. If the second PCR failed, the undiluted PCR-product was used as template. Both PCR reactions were performed in 25 µL triplicate reactions containing 12.5 µL of GoTaq Green Mastermix (Promega, Madison, WI, USA), 25 μM of each primer and 1–2 µL template DNA.

The thermal profile was as follows: Initial denaturation period of 5 min at 94 °C followed by 20 cycles of 94 °C for 30 s, 53 °C (primer pair 1)/58 °C (primer pair 2) for 30 s, 72 °C for 1 min and a final elongation step at 72 °C for 10 min. Triplicate PCR products were pooled together, purified with an Agencourt AMPure XP kit (Beckman Coulter, Krefeld, Germany) and then used as templates for Index PCR (Nextera XT Library Preparation Kit, Illumina, San Diego, CA, USA). The thermal profile was as follows: Initial denaturation at 95 °C for 3 min, 8 cycles of denaturation at 98 °C for 30 s, annealing at 55 °C for 30 s, followed by elongation at 72 °C for 30 s, and a final extension at 72 °C for 5 min. After bead purification and quantification using PicoGreen (Molecular Probes, Eugene, OR, USA), amplicons were pooled in equimolar amounts. A final quality control of this pool was performed using an Agilent 2100 Bioanalyzer (Agilent Technologies, Palo Alto, CA, USA). This amplicon library was used for 2 × 300 bp paired-end sequencing (MiSeq Reagent kit v3) on an Illumina MiSeq system at the Department of Soil Ecology of the Helmholtz-Centre for Environmental Research—UFZ in Halle (Saale), Germany.

### 2.3. Bioinformatics

Amplicon sequencing data were processed using DADA2 [41] implemented in dadasnake [42]. DADA2 uses an error model to identify sequencing errors and resolve exact amplicon sequence variants (ASVs) without involving sequence clustering with an arbitrary cut off. Therefore, this procedure generally identifies fewer, but more reliable units. First, raw reads were searched for both primer sites and primer sequences were cut using cutadapt v1.18 [43]. Only reads with forward and reverse primers were further processed using the DADA2 package in R [44]. Forward and reverse reads were cut to a minimum base quality of nine. Reads with higher expected error rates (maxEE) than three were discarded. Read pairs were merged with an overlap of 20 nt and one mismatch was allowed. Chimera removal was performed using the consensus algorithm. Subsequently, only sequences of 200–450 bp were kept for the analysis. Taxonomic assignment was performed using blastn against the NCBI non-redundant nucleotide sequence database that includes all GenBank, EMBL, DDBJ and PDB sequences, but no environmental samples or metagenomes or unidentified organisms. The taxonomy of each nematode ASV was manually verified down to the family level using the NCBI Taxonomy browser and respective feeding types were assigned according to Yeates, et al. [23] using the online web tool ‘NINJA’ [45]. Based on the family level, six categories were defined: bacterivores, fungivores, herbivores, omnivores, animal parasites, and predators.

### 2.4. Environmental Factors

Wood physico-chemical data (pH, water content, C/N ratio and Klason lignin) and ergosterol as indicator for fungal biomass were taken from Noll, et al. [46] and Moll, et al. [39]. Fungal and prokaryotic α-diversities were reported as observed number of OTUs (operational taxonomic units) of rarefied data sets as applied by Moll, et al. [39] and Leonhardt, et al. [38], respectively. Accordingly, respective β-diversity analyses were based on relative abundances (i.e., sequence counts in each column were scaled by the column’s sum) and subsequent fourth root transformation.

### 2.5. Statistical Analyses

Statistical analyses were performed in R Version 4.0.2 [44] using the interface RStudio (Version, RStudio Inc., Boston, MA, USA). First, the number of sequences per sample and their taxonomic composition at the phylum level were analyzed using the packages “phyloseq” and “microbiome” [47,48]. Some samples contained only few nematode sequences: one *Acer* sapwood sample (AH022_sap), one *Fraxinus* sapwood sample (ES054), two *Populus* heartwood samples (PA023_heart + PA055_heart) and one *Carpinus* heartwood sample (HBU021_heart) (Figure S1). These samples were removed and the remaining dataset was rarefied 1000 times using the command “rarefy_even_depth” to the lowest depth of 858 sequences resulting in saturation of rarefaction curves for all samples (Figure S2). To make results robust against sub-sampling effects, a mean ASV-table of all rarefied versions was used for statistical analyses. Values were fourth root transformed to reduce data range and thus the impact of highly abundant ASVs. 

Nematode community structure related to host tree identity was analyzed separately for sapwood and heartwood by principal coordinates analysis (PCoA) based on Bray-Curtis distance using the function “cmdscale”. Analyses of sapwood samples were performed without the *Fraxinus* sample set and heartwood samples were analyzed without *Carpinus* and *Populus* sample sets to meet three replicates for each category. Permutational multivariate analysis of variance (PerMANOVA) was performed to explore nematode community structure in relation to (a) wood parameters (pH, water content, Klason lignin and C/N ratio) and (b) biotic factors, i.e., (fungal biomass (ergosterol), fungal and prokaryotic α-diversity) based on 999 permutations using the function “adonis” of the “vegan” package [49].

The relationship between nematode community structure and fungal and prokaryotic β-diversity was assessed by Procrustes analyses of PCoA scores based on Bray-Curtis distance and 999 permutations using the “protest” function of the “vegan” package. Thereby, the prokaryotic or fungal ordinations were rotated and scaled to maximum similarity with nematode’s ordination, and a correlation-like statistic (Procrustes R^2^) and the sum of squared differences are reported. 

Nematode α-diversity was defined as observed number of nematode ASVs per rarefied sample. In order to test the relationship between nematode α-diversity and (a) wood parameters and (b) prokaryotic and fungal α-diversities, respectively, Spearman’s rank correlations were performed.

Plots were visualized using “ggplot2” [50] and “patchwork” [51] and partly modified using CorelDRAW^®^ Graphics Suite X8 (Corel Corporation, Ottawa, ON, Canada).

## 3. Results

### 3.1. Sequence Data at a Glance

**Figure 1:**
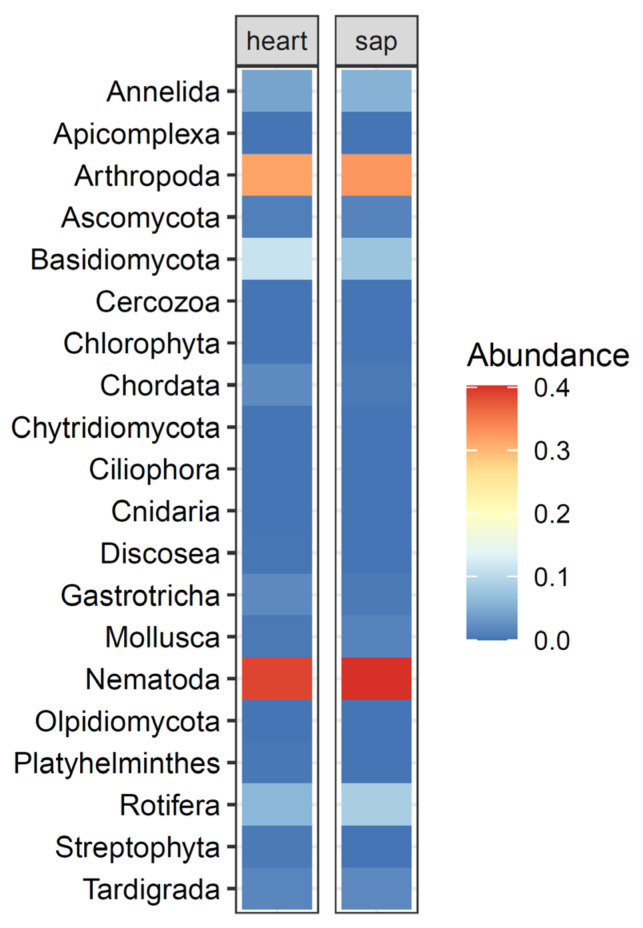
Relative abundances of observed phyla in sapwood and heartwood over all deadwood tree species.

A total of 3,641,045 forward and reverse reads were processed using the dadasnake pipeline. This resulted in 3,215,964 quality filtered reads, which clustered into 663 ASVs. Sequence numbers per sample ranged from 15,445 up to 53,600 (Figure S1). Overall, 39% of all filtered sequences were assigned to the phylum Nematoda. These were clustered into 247 ASVs ranging from 2 up to 25 per deadwood sample. The ratio of sequences taxonomically assigned to nematodes varied strongly from 0% up to 99.99% across samples (Figure S1), but the average ratio was similar between sapwood (40%) and heartwood (39%) (Figure 1). Other frequently observed phyla were Arthropoda, mainly Insecta, Collembola and Arachanida, which comprised 31% of all sequences. The fungal phyla Ascomycota and Basidiomycota accounted together for 11%, Rotifera for 7% and Annelida comprised 5% of the entire data set (Figure 1).

### 3.2. Spatial Community Patterns of Nematodes

Analysis of the rarefied nematode data set excluding non-target sequences revealed nine different nematode orders. Rhabditida dominated with 73% of sequences in the sapwood and 77% in the heartwood followed by Plectida accounting for 19% and 16%, respectively. Dorylaimida accounted for 5% in the sapwood and 3% in the heartwood, respectively. Finally, Triplonchida accounted for 1% in both compartments, while all other orders contributed for less than 1% in total (Figure S3). 

**Figure 2:**
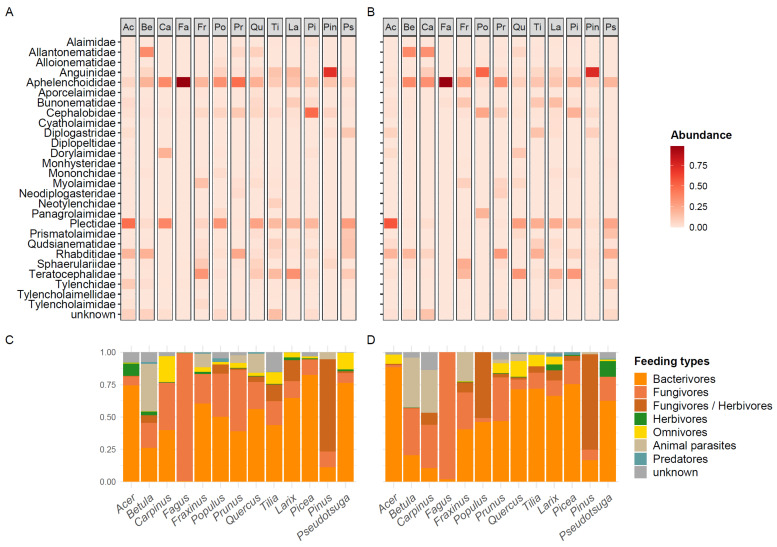
Relative abundances of nematode families visualized by heatmaps (**A**,**B**) and feeding types visualized by stacked bar graphs (**C**,**D**) in sapwood (**A**,**C**) and heartwood (**B**,**D**) of 13 deadwood tree species. *Fraxinus* sapwood n = 2, *Carpinus* heartwood n = 2 and *Populus* heartwood n = 1.

Furthermore, 97% of all nematode sequences could be assigned to the family level representing 27 different families (Figure 2A,B). The majority of these families were identified as free-living according to the ‘Ninja’ data base. In both wood compartments, Aphelenchoididae (sapwood: 26%/heartwood: 24%), Plectidae (18/14%), Rhabditidae (8/10%), Anguinidae (9/13%), Teratocephalidae (8/8%) and Allantonematidae (4/6%) were the most dominant families. Their composition greatly differed between tree species at this taxonomic rank (Figure 2). The majority of nematode families were classified as bacterial (sapwood 48%/heartwood 48%) and fungal feeders (27%/24%) in both wood compartments (Figure 2C,D). About 6% in sapwood and 8% in heartwood were identified as animal parasites followed by 5% and 3% omnivores.

### 3.3. Nematode Community Structure in Relation to Wood Parameters and Co-Occurring Taxa

**Figure 3:**
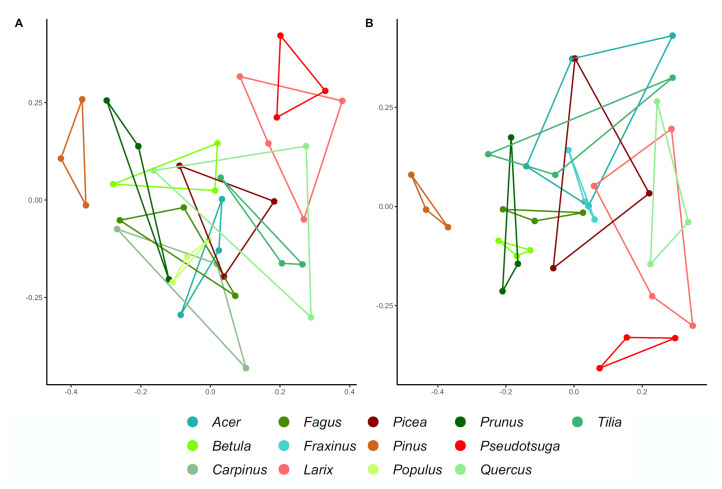
Principal coordinates analysis (PCoA) on Bray-Curtis distance displaying nematode community structure in sapwood (**A**) and heartwood (**B**) in relation to host tree identity (green = deciduous, red = coniferous).

Principal coordinates analysis (PCoA) revealed distinct nematode communities in relation to host tree identity (Figure 3). A PerMANOVA model confirmed the significant differences between nematode community structures of deadwood hosts in both compartments (Table 1). The only wood physico-chemical parameter which significantly contributed to explaining nematode community structure was pH value. However, the presence of co-occurring taxa corresponded to the observed variation. In particular, ergosterol as indicator for fungal biomass, significantly correlated to nematode community variation in both compartments (Table 1). Prokaryotic and fungal α-diversities significantly co-varied with nematode community structure, although only marginally significantly for fungi (Table 1). The link between wood-inhabiting nematodes and fungi as well as prokaryotes sharing the same habitat was further supported by a Procrustes analysis. A strong significant relationship was observed in both wood compartments (all R^2^ > 0.85, *p* < 0.01) using this approach (Table 2, Figure S4). 

**Figure 4:**
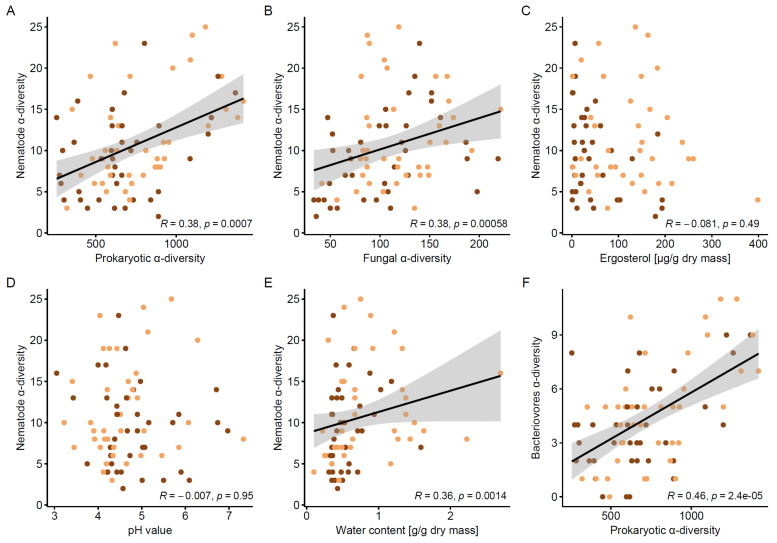
Correlation between nematode α-diversity and (**A**) prokaryotic α-diversity, (**B**) fungal α-diversity, (**C**) ergosterol, (**D**) pH value, (**E**) water content and (**F**) correlation between nematode bacterivore α-diversity and prokaryotic α-diversity. Significance is based on Spearman-rank correlation combined for both wood compartments; light-brown = sapwood, dark-brown = heartwood.

In addition, nematode α-diversity was positively correlated with prokaryotic α-diversity (Spearman’s *ρ* = 0.38, *p* < 0.001), with fungal α-diversity (Spearman’s *ρ* = 0.38, *p* < 0.001) and with water content (Spearman’s *ρ* = 0.36, *p* < 0.001), whereas fungal biomass and pH did not correlate with nematode α-diversity (Figure 4). The correlation between bacterivores α-diversity and that of prokaryotes also resulted in a positive relationship (Spearman’s *ρ* = 0.46, *p* < 0.001), whereas fungivores α-diversity neither correlated with fungal α-diversity nor with fungal biomass (Table S1).

## 4. Discussion

### 4.1. Community Composition of Wood-Inhabiting Nematodes

Overall 247 ASVs from 27 nematode families of 9 orders were observed in the investigated deadwood logs. The two most abundant orders, Rhabditida and Plectida, accounted for more than 90% of sequences. Several observed dominant families of these orders such as Rhabditidae, Plectidae (both bacterivore) or Aphelenchoididae (fungivore) were previously reported from forest soils [20,52,53]. The latter was especially highlighted as a cosmopolitan family tolerating harsh environments and has been currently described from wood [54,55,56]. 

With respect to the feeding types, the majority of sequences (>70%) were assigned to bacterial and fungal feeders. When including Anguinidae, a family comprising both fungivores and herbivores, they even accounted for more than 80% of sequences. Only a small proportion of about 1.5% were classified as herbivores, all assigned to the family Tylenchidae. Members of this family are mainly associates of algae, mosses, lichens or plant roots and have been recovered from forest soils and litter [23,57]. Additionally, fungal feeders, such as *Filenchus* spp., were also described within this family [58]. Animal parasites accounted for approximately 7% of all nematode sequences. The detected families, Allantonematidae and Sphaerulariidae, are known as insect parasites [23], for instance, Scolytinae, which are common inhabitants of wood [59]. Therefore, all detected feeding strategies are plausible within the deadwood habitat as the availability of respective resources such as fungi, bacteria, mosses, algae, lichens or bark beetles can be assumed.

As nematodes were detected in deadwood of all 13 tree species, the question arises of how they enter the substrate. It is difficult to evaluate and trace back the origin of the detected nematodes, though it is known that several taxa are associated with insects (e.g., bark or stag beetles) [60,61,62,63]. Other transport mechanisms seem to be wind, water or plants [56,64]. One could also imagine that nematodes reach the deadwood via small soil particles spread by wind, but whether the underlying soil really serves as a source for wood-inhabiting nematodes is speculation. Hence, further studies are necessary to compare nematode communities between deadwood and the surrounding environment (soil), which will allow to conclusions upon shared taxa and those exclusively identified in deadwood.

### 4.2. Host Tree Identity and Related Wood Parameters

Deadwood host tree identity mainly explained nematode community structure in both sap- and heartwood as revealed by PCoA and PerMANOVA. This host tree effect was previously described for wood-inhabiting prokaryotes, fungi, and beetles, revealing distinct and specific communities, especially between coniferous and deciduous trees [37,65,66,67]. Many members of these organism groups actively degrade wood and thus directly rely on it as available nutrient resource, a fact that serves as possible explanation for the strong host tree identity association [37,66,68]. Here, we also observed host tree-related differences for nematode communities, although they belong to higher trophic levels and do not directly depend on wood as source of nutrition. Hence, we assume that community composition of nematodes is a direct feedback of the concomitance of fungi and prokaryotes (compare Section 4.3) in the same habitat.

As the investigated tree species were highly distinguishable by their wood characteristics [39,46], we anticipated significant explanatory power for nematode community structure. Especially pH and water content are parameters that could directly influence the distribution of nematodes in deadwood, since nematodes are bound to “wet” habitat conditions and their collagenous cuticle is rather sensitive towards high proton concentrations causing protein denaturation [69]. Indeed, pH significantly corresponded to community structure and water content was positively related to the number of observed nematode ASVs (nematode α-diversity). Both parameters were identified as prominent predictors for nematode’s distribution from the micro scale up to global scale [56]. Our results are further in line with a study conducted in soil where fungal and bacterial feeders were affected adversely by increasing pH from 4 to 6 [70]. Other characteristics such as lignin content or the ratio of carbon to nitrogen reflecting the quality of the wood was not found to contribute to their distribution. This is not too surprising, considering that nematodes are not actively degrading wood as described above. Hence, our results suggest distinct coarse habitat filtering for nematodes, but also indicate that other factors may contribute to defining their community assembly.

### 4.3. Co-Occurring Taxa

In agreement with our expectations, co-occurring fungi and prokaryotes were significantly linked to nematodes’ distribution patterns, as revealed by Procrustes analysis between nematode and prokaryotic β-diversity and nematode and fungal β-diversity. Although this may partly reflect similar coarse habitat filtering for the different taxa, it also suggests links between these groups. Since nematodes are not involved in primary wood decay it seems reasonable that biotic interactions with other parts of the deadwood community likely play the most important role for their distribution. This was also supported by analyses on respective α-diversities resulting in positive relationships, i.e., the higher the number of prokaryotes and fungi, the higher the number of nematode ASVs. The same relationship could be confirmed for the number of bacterivore nematodes and prokaryotes indicating that community patterns of bacterivore nematodes are mediated by the deadwood-inhabiting bacterial (prokaryotic) community. Our results corresponded well to previous findings on rhizosphere nematode and bacterial communities of an arable soil showing the same positive relationship [71]. In contrast, neither fungal α-diversity nor fungal biomass correlated with the number of fungivore nematodes, but with the variation of the entire nematode community structure. This could mean that deadwood nematodes are not affected by the total number of fungal species and their densities but are rather related to specific fungal species. In addition, nematodes might be indirectly structured by fungi which have been demonstrated to be able to modify the habitat, e.g., change of pH, and thus directly affect the distribution of other organisms—e.g., bacteria [72,73,74]. Hence, besides habitat filtering effects, our results suggest interactions between the investigated organismic groups, which should be in the focus of subsequent analyses.

### 4.4. Methodological Discussion and Cautionary Note

We followed the approaches of Porazinska, et al. [33] and the adjusted semi-nested PCR approach according to Sapkota and Nicolaisen [34]. The latter observed a proportion of 64% nematode sequences for soil samples, while 39% were reached for deadwood in the present study. In another metabarcoding study on soil samples, a proportion of only 2.5% nematode sequences was observed [75]. The authors discussed this low ratio with the used primers, which were in fact the same used in this study. As they did not follow the semi-nested PCR approach by Sapkota and Nicolaisen [34], this adjustment seems to contribute for increased and sufficient nematode sequences from environmental DNA. Moreover, evaluation of different target regions for metabarcoding of nematodes revealed good taxonomic resolution and a broad base of reference sequences for nematode identification, leading to recommendation of the here chosen amplification strategy and sequence data analysis using DADA2 for further research [76,77]. The emerging number of studies within the last several years clearly emphasizes the great opportunities of metabarcoding but also its challenges [78,79]. Although morphological approaches are time-consuming and strongly depend on specialist’s expertise, one must admit that those are usually preferable to metabarcoding approaches, especially due to the possibility to distinguish between dead and alive organisms and to a better quantitatively exploration. Nevertheless, based on our results, amplicon sequencing can be recommended to identify nematodes in the deadwood substrate.

## 5. Conclusions

Although nematodes are not able to directly utilize wood as a resource, our results reveal diverse communities which are associated with this substrate: (i) Nematodes are presented by various families and many different feeding strategies, (ii) their distribution is significantly related to host tree identity and (iii) they appear to be linked to other deadwood-inhabiting biota including fungi and prokaryotes as main drivers of the decomposition process. Hence, the present study provides novel insights into nematode community structure and points to various advantages in assessing multi-trophic diversity via metabarcoding of a targeted microbiome. Nematodes have been shown to be rather underexplored in terms of their participating role in one of most triggering ecosystem processes—the decomposition of (dead)wood. We are aware that this study only provides a snapshot of an early to middle stage of decomposition and is exemplary for one forest site. Nevertheless, we here provide a clear rationale for further research with an emphasis to address open questions, whether nematodes simply act as “passengers” in the system, or if they actively influence the decay process, e.g., by affecting competition and colonization scenarios between primary decomposers.

## Figures and Tables

**Table 1:** Results of PerMANOVA based on Bray-Curtis dissimilarities for nematode community structure in relation to the investigated biotic factors (fungal biomass, fungal and prokaryotic α-diversity), wood physico-chemical properties (pH, water content, Klason lignin, C/N ratio) and host tree identity (tree species); *p*-values were based on 999 permutations; boldface indicates statistical significance with *p* < 0.05, italic marginally significance with *p* < 0.1.

	Sapwood			Heartwood		
	F.Model	R^2^	*p*	F.Model	R^2^	*p*
Fungal biomass	1.48757	0.03610	**0.009**	1.76363	0.04346	**0.004**
Fungal α-diversity	1.34849	0.03272	*0.097*	1.35050	0.03328	*0.084*
Prokaryotic α-diversity	2.19116	0.05317	**0.001**	2.12316	0.05232	**0.002**
pH	1.55533	0.03774	**0.015**	1.70446	0.04200	**0.012**
Water content	1.11823	0.02714	0.295	1.20579	0.02971	0.177
Klason lignin	1.24776	0.03028	0.146	1.07884	0.02659	0.319
C/N ratio	0.99676	0.02419	0.506	0.98509	0.02427	0.501
Tree species	1.20560	0.32183	**0.027**	1.33691	0.32945	**0.004**
Residuals		0.43682			0.41892	
Total		1.00000			1.00000	

**Table 2:** Results of Procrustes analyses between deadwood-inhabiting nematodes and prokaryotes or fungi, respectively; correlations are based on PCoA results using the ‘protest’ function in vegan. Boldface indicates statistical significance with *p* < 0.05. Related plots showing the similarity of correlations are given in supplemental Figure S4.

	Prokaryotes	Fungi	Prokaryotes	Fungi
Procrustes Sum of Squares	0.2728	0.2433	0.243	0.2103
Correlation R^2^	0.8528	0.8699	0.8701	0.8887
*p* value	**0.001**	**0.003**	**0.001**	**0.001**

## Data Availability

All raw sequences have been submitted to the NCBI short read archive (SRA, https://www.ncbi.nlm.nih.gov/sra/) and are accessible under the number PRJNA714549.

## Supplementary Materials

The following are available online at https://www.mdpi.com/article/10.3390/microorganisms9071454/s1, Figure S1: Number of reads and taxonomical assignment at the phylum level for each sample, Figure S2: Rarefaction curves for each sample at rarefied sequencing depth of 858 reads, Figure S3: Relative abundances of nematode orders in sapwood and heartwood of the 13 deadwood tree species investigated. Fraxinus sapwood n = 2, Carpinus heartwood n = 2 and Populus heartwood n = 1, Figure S4: Procrustes superimposition plots showing the degree of correlation between nematodes and prokaryotes (A&C) and nematodes and fungi (B&D) in sapwood (A&B) and heartwood (C&D), Table S1: Spearman’s rank correlation test between bacterivores and prokaryotic α-diversities, between fungivores α-diversity and fungal α-diversity and between fungal biomass (ergosterol) and fungivores α-diversity for both wood compartments and for sapwood and heartwood separately; ns = not significant.
