# Supplementary material for: First Evidence That Nematode Communities in Deadwood Are Related to Tree Species Identity and to Co-Occurring Fungi and Prokaryotes
Source: Microorganisms. 2021 Jul 6;9(7):1454. doi: 10.3390/microorganisms9071454 (PMC8304250; doi:10.3390/microorganisms9071454)
Supplement: Supplementary file 1 [file microorganisms-09-01454-s001.zip › Moll et al 2021_Fig.S4.pdf]

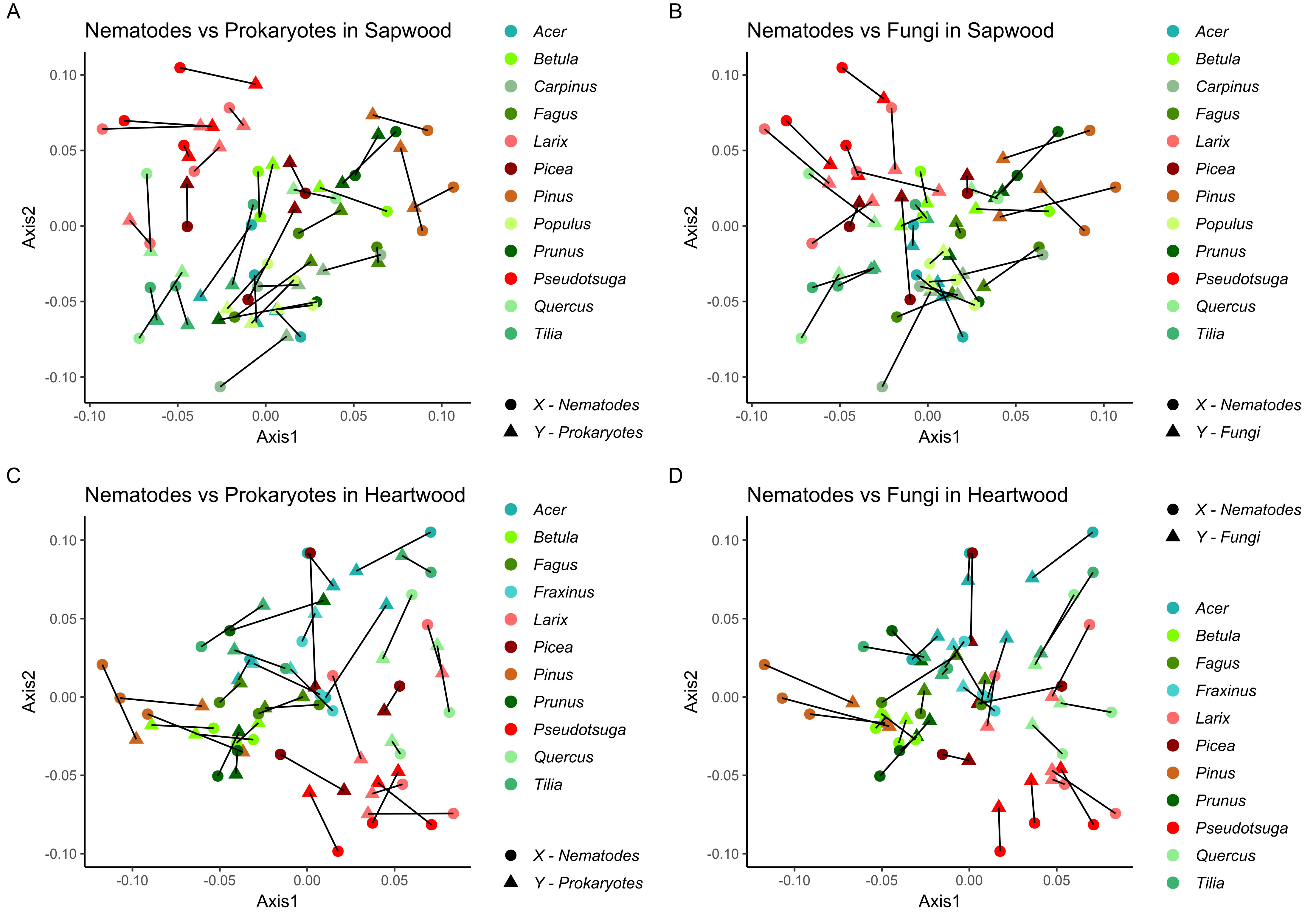
A
Nematodes vs Prokaryotes in Sapwood
B
Nematodes vs Fungi in Sapwood
C
Nematodes vs Prokaryotes in Heartwood
D
Nematodes vs Fungi in Heartwood
Axis1
Axis2
Acer
Betula
Carpinus
Fagus
Fraxinus
Larix
Picea
Pinus
Populus
Prunus
Pseudotsuga
Quercus
Tilia
X - Nematodes
Y - Prokaryotes
Y - Fungi
