# Supplementary material for: First Evidence That Nematode Communities in Deadwood Are Related to Tree Species Identity and to Co-Occurring Fungi and Prokaryotes
Source: Microorganisms. 2021 Jul 6;9(7):1454. doi: 10.3390/microorganisms9071454 (PMC8304250; doi:10.3390/microorganisms9071454)
Supplement: Supplementary file 1 [file microorganisms-09-01454-s001.zip › Moll et al. 2021_Table S1.pdf]

Supporting information

First evidence that nematode communities in deadwood are related to tree species identity and to co-occurring fungi and prokaryotes

Julia Moll, Friederike Roy, Claus Bässler, Jacob Heilmann-Clausen, Martin Hofrichter, Harald Kellner, Doris Krabel, Jan Henrik Schmidt, François Buscot, Björn Hoppe

Table S1: Spearman's rank correlation test between bacterivores and prokaryotic α-diversities, between fungivores α-diversity and fungal α-diversity and between fungal biomass (ergosterol) and fungivores α-diversity for both wood compartments and for sapwood and heartwood separately; ns = not significant.

	Both	Sapwood	Heartwood
	Prokaryotic α-diversity *vs.* bacterivores α-diversity		
Spearman's ρ	0.4614347	0.5592075	0.3331085
P value	**2.403e-05**	**0.0002151**	**0.04099**
	Fungal α-diversity *vs.* fungivores α-diversity		
Spearman's ρ	0.1298779	0.05293589	0.1839097
P value	ns	ns	ns
	Fungal biomass (ergosterol) *vs.* fungivores α-diversity		
Spearman's ρ	-0.04781332	-0.1410455	-0.09970198
P value	ns	ns	ns
