# Supplementary figures and images for: First Evidence That Nematode Communities in Deadwood Are Related to Tree Species Identity and to Co-Occurring Fungi and Prokaryotes
Source: Microorganisms. 2021 Jul 6;9(7):1454. doi: 10.3390/microorganisms9071454 (PMC8304250; doi:10.3390/microorganisms9071454)

## Sapwood

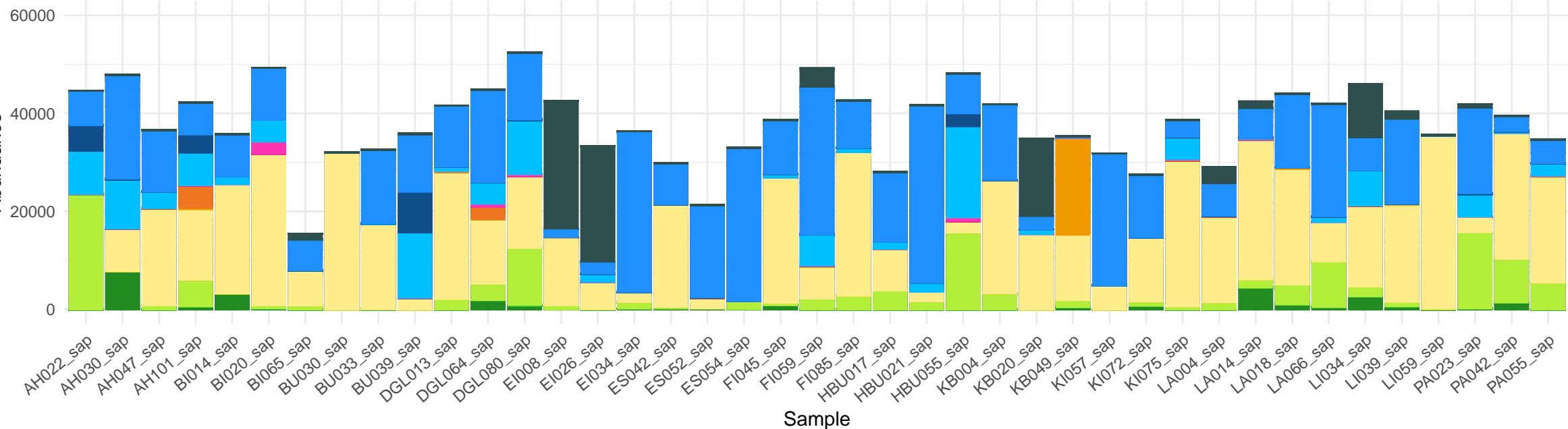

## Heartwood

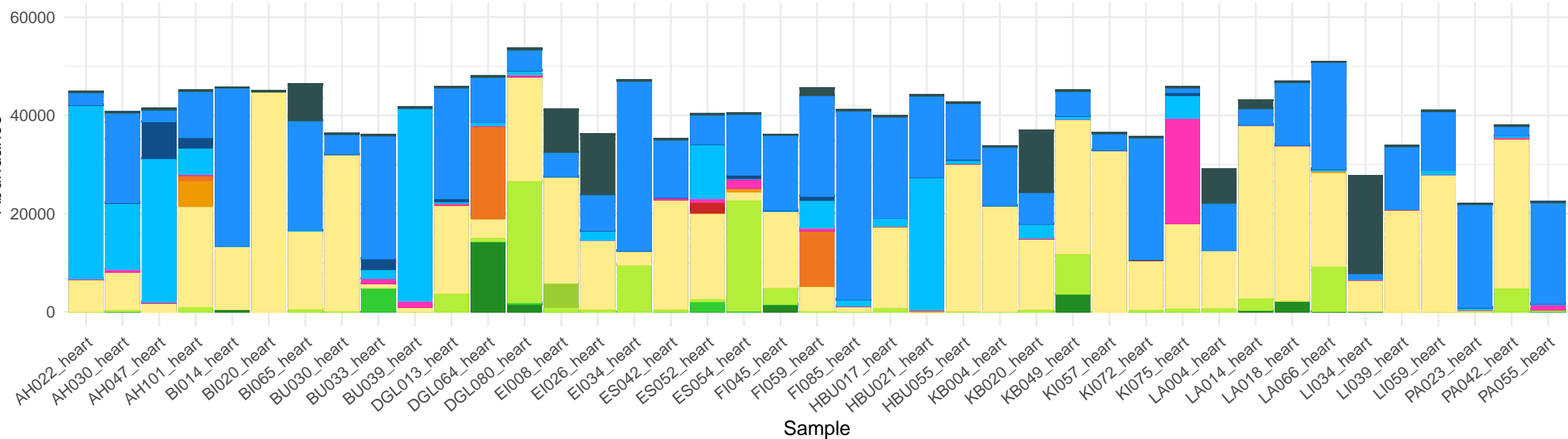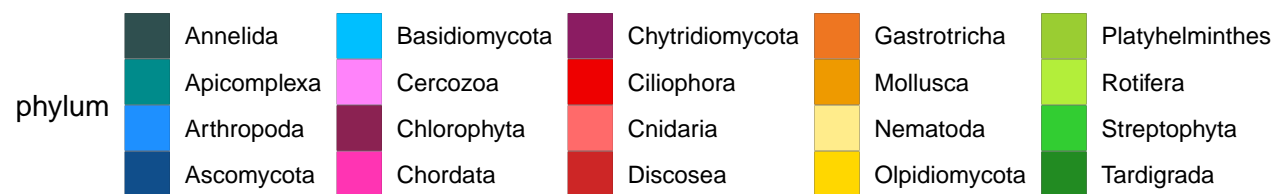

Supplement: Supplementary file 1 [file microorganisms-09-01454-s001.zip › Moll et al 2021_Fig.S1.pdf]

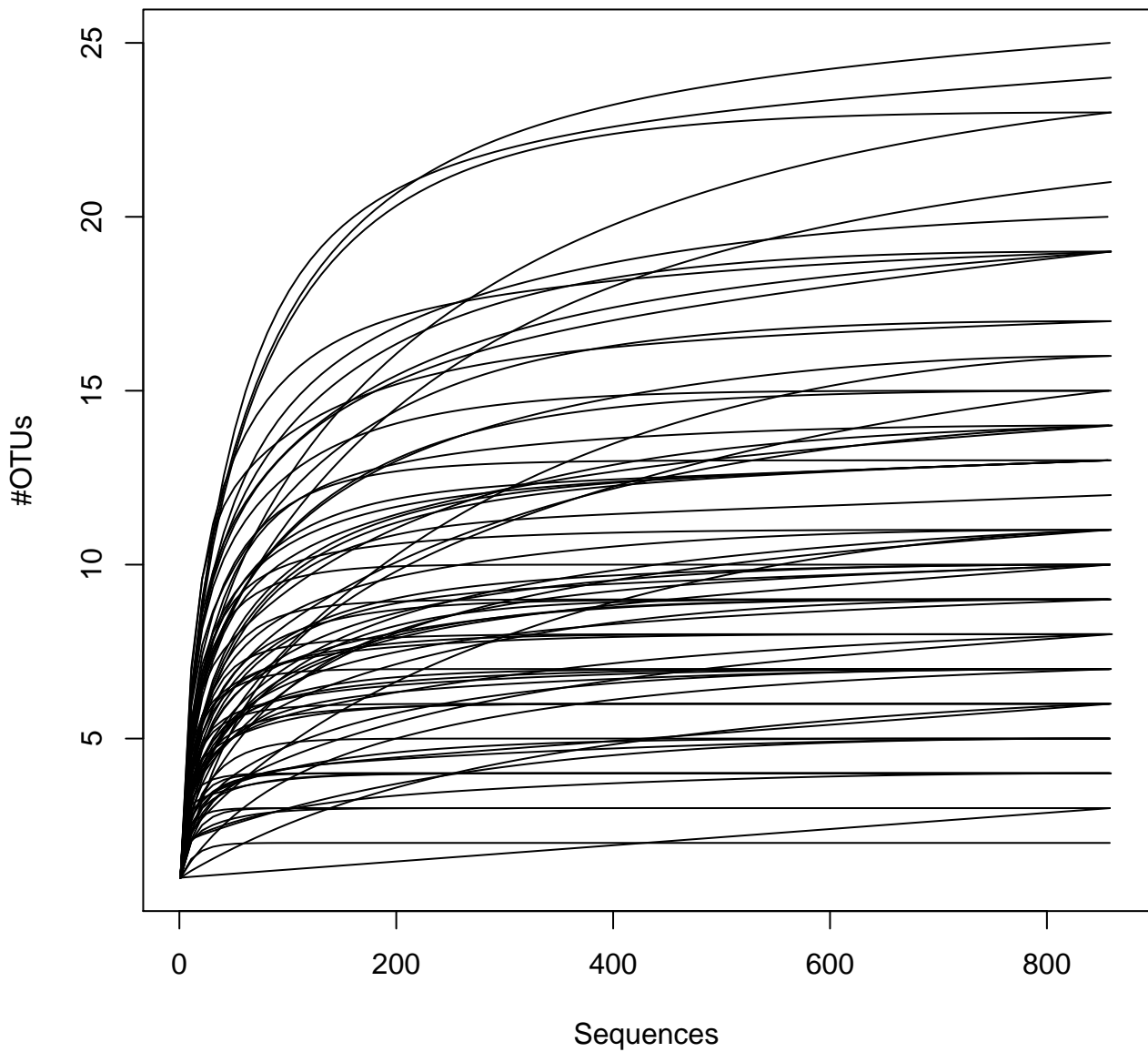

Supplement: Supplementary file 1 [file microorganisms-09-01454-s001.zip › Moll et al 2021_Fig.S2.pdf]

## Sapwood

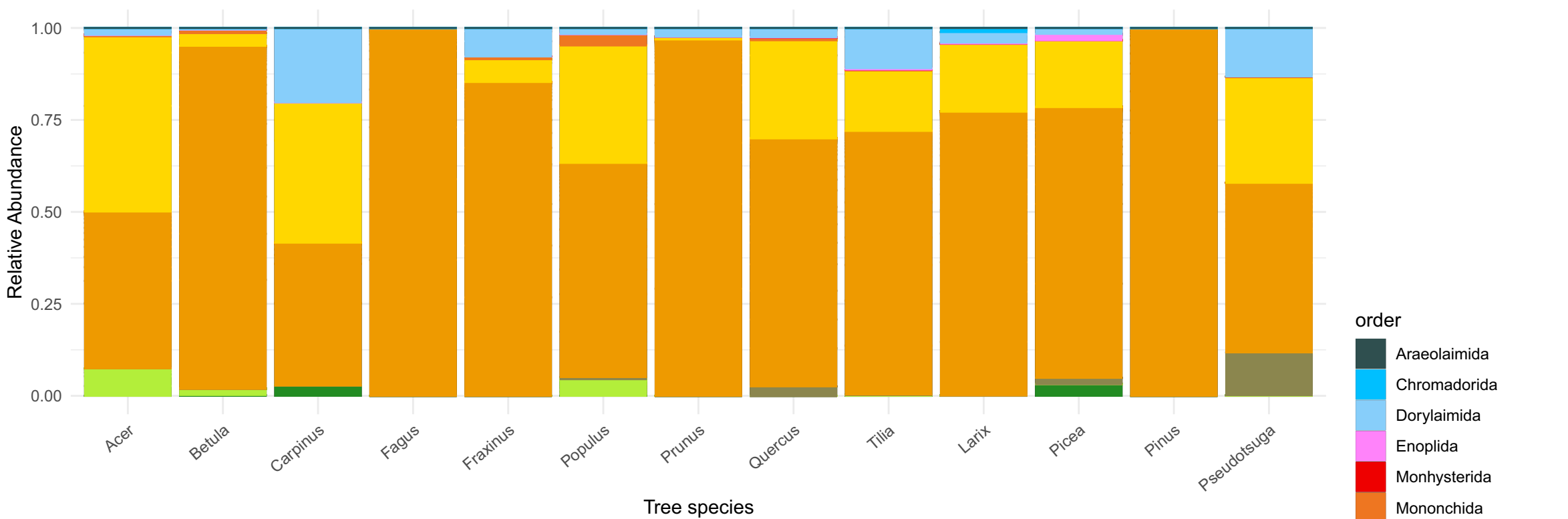

## Heartwood

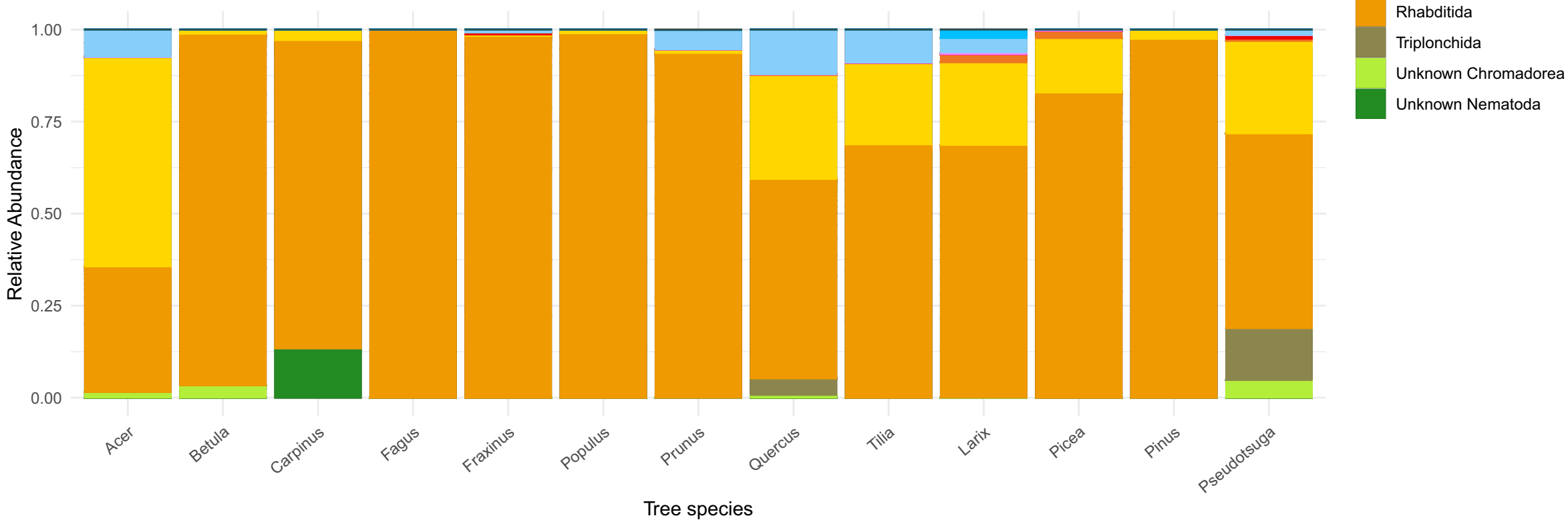

Supplement: Supplementary file 1 [file microorganisms-09-01454-s001.zip › Moll et al 2021_Fig.S3.pdf]
